# Potato tuberization under long-day conditions: Genetic effects of 6 *CYCLING DOF FACTOR1* alleles

**DOI:** 10.1093/plphys/kiaf098

**Published:** 2025-03-27

**Authors:** Lianlian Ma, Haicai Li, Hao Jiang, Yanhui Zhu, Zhong Zhang, Dawei Li, Guangtao Zhu, Qijun Sui, Yinqiao Jian, Jianjian Qi, Zefeng Zhai, Chunzhi Zhang

**Affiliations:** Shenzhen Branch, Guangdong Laboratory of Lingnan Modern Agriculture, Key Laboratory of Synthetic Biology, Ministry of Agriculture and Rural Affairs, Agricultural Genomics Institute at Shenzhen, Chinese Academy of Agricultural Sciences, Shenzhen 518120, China; College of Agriculture, South China Agriculture University, Guangzhou 510642, China; Shenzhen Branch, Guangdong Laboratory of Lingnan Modern Agriculture, Key Laboratory of Synthetic Biology, Ministry of Agriculture and Rural Affairs, Agricultural Genomics Institute at Shenzhen, Chinese Academy of Agricultural Sciences, Shenzhen 518120, China; Shenzhen Branch, Guangdong Laboratory of Lingnan Modern Agriculture, Key Laboratory of Synthetic Biology, Ministry of Agriculture and Rural Affairs, Agricultural Genomics Institute at Shenzhen, Chinese Academy of Agricultural Sciences, Shenzhen 518120, China; Shenzhen Branch, Guangdong Laboratory of Lingnan Modern Agriculture, Key Laboratory of Synthetic Biology, Ministry of Agriculture and Rural Affairs, Agricultural Genomics Institute at Shenzhen, Chinese Academy of Agricultural Sciences, Shenzhen 518120, China; Shenzhen Branch, Guangdong Laboratory of Lingnan Modern Agriculture, Key Laboratory of Synthetic Biology, Ministry of Agriculture and Rural Affairs, Agricultural Genomics Institute at Shenzhen, Chinese Academy of Agricultural Sciences, Shenzhen 518120, China; Yunnan Key Laboratory of Potato Biology, The AGISCAAS-YNNU Joint Academy of Potato Sciences, Yunnan Normal University, Kunming 650000, China; Shenzhen Branch, Guangdong Laboratory of Lingnan Modern Agriculture, Key Laboratory of Synthetic Biology, Ministry of Agriculture and Rural Affairs, Agricultural Genomics Institute at Shenzhen, Chinese Academy of Agricultural Sciences, Shenzhen 518120, China; State Key Laboratory of Vegetable Biobreeding, Institute of Vegetables and Flowers, Chinese Academy of Agricultural Sciences, Beijing 100081, China; Inner Mongolia Potato Engineering and Technology Research Center, Key Laboratory of Herbage and Endemic Crop Biology, Ministry of Education, School of Life Sciences, Inner Mongolia University, Hohhot 010021, China; Shenzhen Branch, Guangdong Laboratory of Lingnan Modern Agriculture, Key Laboratory of Synthetic Biology, Ministry of Agriculture and Rural Affairs, Agricultural Genomics Institute at Shenzhen, Chinese Academy of Agricultural Sciences, Shenzhen 518120, China; Shenzhen Branch, Guangdong Laboratory of Lingnan Modern Agriculture, Key Laboratory of Synthetic Biology, Ministry of Agriculture and Rural Affairs, Agricultural Genomics Institute at Shenzhen, Chinese Academy of Agricultural Sciences, Shenzhen 518120, China

## Abstract

Analysis of 6 *CYCLING DOF FACTOR1* alleles reveals allele-specific adaptation to high- and low-latitude environments, providing a framework for breeding diploid potato for long-day conditions.

Dear Editor,

Potato (*Solanum tuberosum* L.) is the most important tuber crop globally (Stokstad 2019). However, tetrasomic inheritance-related complexities limit the genetic improvement of tetraploid cultivars. Considerable efforts have been directed toward utilizing seed-propagated inbred line-based diploid potatoes (Jansky et al. 2016; Zhang et al. 2021). However, given that the tuberization of most naturally occurring diploid potato germplasm essentially depends on a short-day photoperiod, the development of inbred diploid lines with long-day adaptability remains challenging.

The identification of *StCDF1* has paved the way for improving long-day adaptation in diploid potatoes (Kloosterman et al. 2013). Mediated by FLAVIN-BINDING, KELCH REPEAT, F-BOX (StFKF1)/GIGANTEA (StGI), the wildtype allele, *StCDF1.1* is ubiquitinated and subsequently degraded, liberating *CONSTANS (StCO)* and suppressing the expression of the tuberigen *SELF PRUNING 6A* (*StSP6A)*. Conversely, mutated alleles, *StCDF1.2* and *StCDF1.3*, inhibit *StCO* expression, thereby upregulating *StSP6A* and promoting tuberization under long-day conditions (Navarro et al. 2011; Kloosterman et al. 2013; Abelenda et al. 2016).

To identify optimal alleles for improving diploid potato landraces, we examined allelic variations in *StCDF1* and evaluated the genetic effects of its alleles. Specifically, we resequenced 64 tetraploid cultivars, 10 wild *Solanum candolleanum* accessions, and 10 diploid landraces (Supplementary Table S1). The 64 cultivars account for approximately 60% of the total cultivated area in China. The phylogenetic tree, population structure, and principal component analyses supported the genetic divergence among the tetraploid cultivars, wild accessions, and diploid landraces (Supplementary Fig. S1, A and B and Fig. 1A). For tetraploid cultivars, the northern- and southern-adapted varieties clustered separately, suggesting latitude-dependent genetic differentiation (Fig. 1A).

**Figure 1.**
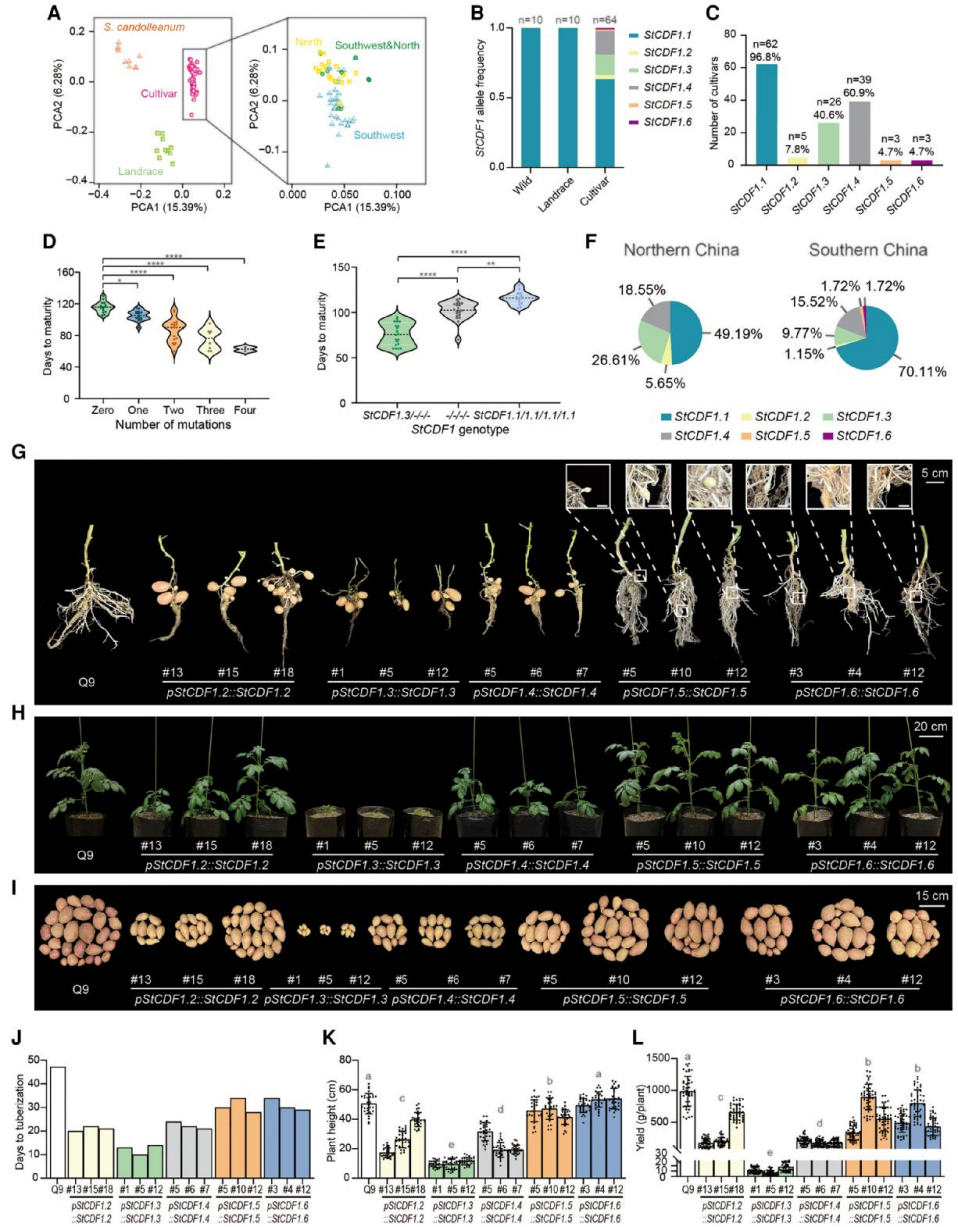
Genetic effects of *StCDF1* alleles on tuberization under long-day conditions, and their breeding applications. **A)** Left: Principal component analysis (PCA) of 84 potato accessions. The proportions of variance explained by the principal components are presented in the axis labels. Right: PCA of potato cultivars (boxed region, left panel). **B)** Allele frequencies of 6 *StCDF1* alleles in wild, landrace, and cultivar plants. **C)** Number of cultivars with each of the 6 *StCDF1* alleles among 64 potato cultivars. **D)** Relationship between mutant *StCDF1* allele copy number and growth period. Dunnett's T3 multiple comparisons test, *P < 0.05, **P < 0.01, ***P < 0.001, ****P < 0.0001. **E)** Relationship between *StCDF1* genotype and days-to-maturity. *StCDF1.3/−/−/−*, genotypes including *StCDF1.3*; *−/−/−/−*, genotypes excluding *StCDF1.3*; *StCDF1.1/1.1/1.1/1.1*, genotype in which all 4 copies are *StCDF1.1*; Dunnett's T3 multiple comparisons test, *P < 0.05, **P < 0.01, ***P < 0.001, ****P < 0.0001. **F)** Latitudinal distribution of 6 *StCDF1* alleles in China. **G–H**) Tuberization and plant height of *StCDF1* transgenic lines after cultivation for 45 d under artificial long-day conditions (16 h light/8 h dark). Images were digitally extracted for comparison. The scale bar of the white rectangle = 1 cm. **I)**  *StCDF1* transgenic-line tubers at maturity under natural long-day conditions in Inner Mongolia (*n* = 3 plants). Images were digitally extracted for comparison. **J–L**) *StCDF1* transgenic-line tuber initiation time, plant height (*n* = 30 plants), and yield (*n* = 50 plants) under natural long-day conditions in Inner Mongolia. The results are presented as means ± SD. Tukey's multiple comparisons test, alpha = 0.05.

The analysis of time-to-maturity for the 64 cultivars identified the *StCDF1* locus on chromosome 5 as a key player in photoperiodic adaptation (Supplementary Fig. S2; Supplementary Table S1). Resequencing revealed 6 *StCDF1* alleles, including 2 new alleles: *StCDF1.5* and *StCDF1.6* (Supplementary Figure S3, A to C; Supplementary Table S2). Allelic frequency analysis revealed all *StCDF1* mutants in tetraploid varieties; however, wild accessions and landraces sequenced in this study only contained wildtype *StCDF1.1* (Figure 1B). Furthermore, 96.8%, 60.9%, and 40.6% of the cultivars contained at least one copy of *StCDF1.1*, *StCDF1.4*, and *StCDF1.3*, respectively (Fig. 1C). Although both *StCDF1.2* and *StCDF1.4* harbor a 7-bp insertion at the same position (Gutaker et al. 2019), *StCDF1.2* was observed only in 5 accessions (Fig. 1C). Furthermore, *StCDF1.5* and *StCDF1.6* exhibited the lowest frequency, each occurring only in 3 cultivars (Fig. 1C). Mutant allele copy number was negatively associated with time-to-maturity (Fig. 1D). Cultivars containing *StCDF1.3* exhibited the shortest time-to-maturity (Fig. 1E), which is consistent with previous studies (Caraza-Harter and Endelman 2022; Hoopes et al. 2022). Moreover, we observed relatively high *StCDF1.2*, *StCDF1.3*, and *StCDF1.4* frequencies in cultivars from northern China, whereas *StCDF1.1*, *StCDF1.5*, and *StCDF1.6* showed relatively high frequencies in cultivars from the low-latitude southwestern region (Fig. 1F). We hypothesize that *StCDF1.2–1.4* confer strong adaptability to long-day conditions, whereas *StCDF1.1*, *StCDF1.5*, and *StCDF1.6* might be appropriate for low-latitude environments.

We analyzed the genetic effects of *StCDF1* alleles by transforming the full-length sequences of *StCDF1.2–1.6*, under their native promoters, into the Qingshu9 background (*StCDF1.1/1.1/1.1/1.1*, respectively) and validated the transgenic lines using corresponding molecular markers (Supplementary Fig. S4, A to C). Under both natural and artificial long-day conditions, tuberization started earliest in the *StCDF1.3* line (Fig. 1, G and J; Supplementary Fig. S5A), which, however, exhibited short plants, thin shoots, small tubers, and low yields (Fig. 1, G to L; Supplementary Fig. S5, A and B), possibly owing to transposon insertion. Tuberization initiation time did not vary substantially between *StCDF1.2* and *StCDF1.4* lines approximately 10 d after the *StCDF1.3* lines (Fig. 1, G and J; Supplementary Fig. S5A). *StCDF1.5* and *StCDF1.6* lines tuberized approximately 20 d after the *StCDF1.3* lines (Fig. 1, G and J; Supplementary Fig. S5A). Although the wildtype Qingshu9 as well as the *StCDF1.5* and *StCDF1.6* lines produced high yields (Fig. 1, I and L), they were harvested 1 month later than other lines. In the normal growing season of northern China, these materials tuberize too late to reach maturity. In addition, evaluating the effects of the *StCDF1* alleles in 4 segregating populations revealed a similar order of tuberization initiation; however, *StCDF1.5* and *StCDF1.6* only slightly accelerate tuberization compared with *StCDF1.1* in such a heterozygous background (Supplementary Fig. S6). In brief, under long-day conditions, *StCDF1.3* exerted the strongest effect on tuberization, followed by *StCDF1.2*/*StCDF1.4*, and *StCDF1.5* and *StCDF1.6*.

To elucidate the molecular effects of *StCDF1* alleles, we analyzed gene expression in the *StCDF1*-mediated pathway (Supplementary Fig. S7A). *StGI* and *StFKF1* were activated by light and regulated by *StCDF1* via an as-yet-uncharacterized feedback pathway, especially in the *StCDF1.3* transgenic lines (Supplementary Fig. S7, B to D). *StCOL1 and StCOL2*, which inhibit tuberization, were directly suppressed by *StCDF1* (Kloosterman et al. 2013; Abelenda et al. 2016). In transgenic lines, *StCOL2* expression was inversely correlated with tuberization initiation time (Supplementary Figs. S5A, S7, E and F, and S8). Although *StCOL1* was activated at dawn, its relative expression level at dusk was weakened in transgenic lines. A similar observation was made for *StCOL2*, suggesting that the peak expression level at dusk plays a key role in regulating tuberization (Supplementary Figs. S7, E and F and S8). Two *FT* family genes, *StSP5G* and *StSP6A*, showed negative and positive correlations with tuberization initiation time, respectively (Supplementary Figs. S5A and S7, G and H), further confirming their maintenance during photoperiod-regulated tuberization.

Through the analysis of gene expression patterns within the *StCDF1* pathway, we hypothesize the differential tuberization effects of *StCDF1.5* and *StCDF1.6*. Compared with *StCDF1.1*, *StCDF1.5* has 2 additional amino acids but exhibits a slight tuberization-promoting effect (Supplementary Figs. S3C and 1G). We observed that the *StCDF1.5* allele was highly expressed compared with other alleles except *StCDF1.3* (Supplementary Fig. S7D), which might explain why *StCDF1.5* expedites earlier tuberization in comparison to *StCDF1.1*. Although *StCDF1.6* lacks the StFKF1-binding domain (similar to *StCDF1.2*, *StCDF1.3*, and *StCDF1.4*), it exhibits a reduced tuberization-promoting effect (Supplementary Fig. S3C and 1G). One possible explanation is that *StCDF1.6* weakens its suppression of *StCOL1/2* (Supplementary Fig. S7, E and F), which indirectly leads to reduced expression of *StSP6A* (Supplementary Fig. S7H), ultimately resulting in a weak tuberization-promoting effect of *StCDF1.6* compared with *StCDF1.2*, *StCDF1.3*, and *StCDF1.4*.

To utilize *StCDF1* in diploid hybrid breeding, we crossed Lishu6 (*StCDF1.1/1.1/1.4/1.5*) and Eshu5 (*StCDF1.1/1.1/1.4/1.6*) with the haploid inducer PL4 (*StCDF1.1/1.1*). From the progeny, we obtained dihaploids containing *StCDF1.4, StCDF1.5*, or *StCDF1.6* (Supplementary Fig. S9, A to F), highlighting opportunities for applying long-day adaptation genes in tetraploid lines in diploid breeding.

In summary, we identified 6 *StCDF1* alleles (*StCDF1.1–1.6*) involved in photoperiod-regulated tuberization. *StCDF1.2* and *StCDF1.4* provided a balance between tuberization and yield under long-day conditions, suggesting that they are optimal for improving long-day adaptability. Additionally, *StCDF1.4*−, *StCDF1.5*−, and *StCDF1.6*-containing dihaploids may serve as germplasm resources for breeding long-day-adapted diploid potatoes. Combining various *StCDF1* alleles may also be useful for maximizing yield adapted to particular latitudes.

## Supplementary Material

kiaf098_Supplementary_Data

## Data Availability

The whole-genome sequencing data are deposited at the National Center for Biotechnology Information with Sequence Read Archive accession numbers SUB14817678 and SUB14886444.

## Supplementary data

The following materials are available in the online version of this article.

**Supplementary Figure S1.** Genomic diversity and population structure of the 84 potato accessions used in this study.

**Supplementary Figure S2.** Genome-wide association study of the day-to-maturity using 64 potato cultivars.

**Supplementary Figure S3.** Gene structure and sequences of *StCDF1.1–1.6*.

**Supplementary Figure S4.** Identification of *StCDF1* transgenic lines.

**Supplementary Figure S5.** Phenotypic analysis of *StCDF1* transgenic lines in artificial long-day conditions.

**Supplementary Figure S6.** Effects of *StCDF1* alleles on tuberization initiation timing in a tetraploid heterozygous background.

**Supplementary Figure S7.** Expression of genes related to the *StCDF1*-mediated photoperiod-regulated tuberization pathway.

**Supplementary Figure S8.** Diurnal expression of *StCOL1* and *StCOL2* in the *StCDF1* transgenic lines under long-day conditions.

**Supplementary Figure S9.** Generation of long-day-adapted dihaploid lines containing *StCDF1.4–1.6* through haploid induction technology.

**Supplementary Table S1.** Details on 84 studied accessions.

**Supplementary Table S2.** Reads support for different *StCDF1* alleles.

**Supplementary Table S3.** Primers used in this study.
