# Supplementary material for: Potato tuberization under long-day conditions: Genetic effects of 6 CYCLING DOF FACTOR1 alleles
Source: Plant Physiol. 2025 Mar 27;197(3):kiaf098. doi: 10.1093/plphys/kiaf098 (PMC11950725; doi:10.1093/plphys/kiaf098)
Supplement: kiaf098_Supplementary_Data [file kiaf098_supplementary_data.zip › Supplementary data.docx]

**Potato tuberization under long-day conditions: Genetic effects of six CYCLING DOF FACTOR1 alleles**

Lianlian Ma^1^, Haicai Li^2^, Hao Jiang^1^, Yanhui Zhu^1^, Zhong Zhang^1^, Dawei Li^1^, Guangtao Zhu^3^, Qijun Sui^1^, Yinqiao Jian^4^, Jianjian Qi^5^, Zefeng Zhai^1,^*, Chunzhi Zhang^1,^*

^1^Shenzhen Branch, Guangdong Laboratory of Lingnan Modern Agriculture, Key Laboratory of Synthetic Biology, Ministry of Agriculture and Rural Affairs, Agricultural Genomics Institute at Shenzhen, Chinese Academy of Agricultural Sciences, Shenzhen, China

^2^College of Agriculture, South China Agriculture University, Guangzhou, China

^3^Yunnan Key Laboratory of Potato Biology, The AGISCAAS-YNNU Joint Academy of Potato Sciences, Yunnan Normal University, Kunming, China

^4^State Key Laboratory of Vegetable Biobreeding, Institute of Vegetables and Flowers, Chinese Academy of Agricultural Sciences, Beijing, China

^5^Inner Mongolia Potato Engineering and Technology Research Center, Key Laboratory of Herbage and Endemic Crop Biology, Ministry of Education, School of Life Sciences, Inner Mongolia University, Hohhot, China

*Correspondence should be addressed to Chunzhi Zhang (zhangchunzhi01@caas.cn) or Zefeng Zhai (zhaizefeng@126.com)

**Supplementary Materials and Methods**

**Plant materials and growth conditions.**

The 10 wild accessions and 10 diploid landraces used in this study were kindly provided by the International Potato Center. The genotypes GS428, BS280, and BS287, used for cloning *StCDF1.2*, *StCDF1.3*, and *StCDF1.4*, respectively, were obtained from the USDA. For both *StCDF1*-transformed and non-transformed control plants, in vitro plantlets were grown in a growth chamber (BPC500H, Fujian Jiupo Biotechnology Co., Ltd, China) under LD conditions, then transfered to greenhouse after 2 weeks. And mini-tubers were planted in Hohhot, Inner Mongolia (40° 50′ N, 111° 44′ E) under natural long days. The phenotypes we evaluated include days to tuberization, plant height, and tuber yield. Days to tuberization is defined as the time from seedling emergence or tissue-cultured seedlings are transplanted into the soil until the stolons begin to swell.

To evaluate the effects of different *StCDF1* genotypes using segregating populations, crosses were made using Zhongshu3 (*StCDF1.1/1.1/1.3/1.4*), Jizhangshu12 (*StCDF1.1/1.1/1.2/1.4*), Lishu6 (*StCDF1.1/1.1/1.4/1.5*), and Eshu5 (*StCDF1.1/1.1/1.4/1.6*) as maternal parents and C88 (*StCDF1.1/1.1/1.1/1.1*) as the paternal parent. The *StCDF1* genotypes containing a single mutant allele copy were identified by molecular markers, and tubers were planted under long-day conditions in the greenhouse (16 h light/8 h dark). Tuberization rate was evaluated weekly, beginning three weeks after seedling emergence, by monitoring the number of plants initiating tuberization out of ten per genotype. The tuberization rate was calculated as the number of plants initiating tuberization divided by ten.

**Identification of *StCDF1* isoform copy numbers and variant calling.**

Leaf DNA was extracted using the cetyl trimethyl ammonium bromide (CTAB) method. A total of 1 μg DNA was used for library construction, followed by sequencing on the DNBSEQ platform. The sequencing depth was 10× for the ten wild accessions and ten diploid landraces and 100× for the 64 cultivars. Paired-end resequencing reads were aligned to the potato reference genome DM1-3 516 R44 (DM) (V6.1) (Pham et al., 2020) using BWA (v0.7.5a-r405) (Li et al., 2009). The generated BAM files were sorted using Samtools (v1.9) (Li et al., 2009), and GATK (v4.2.1.0) was employed to mark duplicate reads (McKenna et al., 2010). The BAM files were visualized using IGV software. *StCDF1* isoforms were determined based on insertions or deletions (indels) in the *StCDF1* gene, and *StCDF1* allele copy numbers were calculated according to the proportion of reads between each *StCDF1* isoform. The specific read counts are listed in Supplementary Table S2. SNPs were extracted using BCFtools (v1.9) (Li et al., 2009). VCFtools was utilized to filter low-quality SNPs using the following parameters: minQ 30, maf 0.05, max-missing 0.7, min-alleles 2, max-alleles 2.

**Population genetic analyses.**

High-quality SNPs were filtered using fourfold degenerate synonymous sites. These SNPs were used to construct a maximum likelihood phylogenetic tree with RAxML (bootstrap 1000) and visualized using the online tool iTOL (https://itol.embl.de). PCA was conducted using these SNPs with Plink (command: pca). The population structure diagram was inferred using Admixture and visualized in R.

**GWAS for potato maturity.**

A total of 8,089,005 high-quality SNPs (MAF > 0.05) were used to perform a genome-wide association study (GWAS) for maturity in 64 potato cultivars. Maturity data was obtained from online resources. Association analyses were conducted using the mixed linear model (MLM) in efficient mixed-model association expedited (EMMAX) software. Kinship was derived from all SNPs. The significance threshold for association was set at 1/n, where n represents the total number of SNPs.

**Plasmid construction and plant transformation.**

The *StCDF1* alelles *StCDF1.2*, *StCDF1.3*, *StCDF1.4*, *StCDF1.5*, and *StCDF1.6* were cloned from GS428 (*StCDF1.1/1.2*), BS280 (*StCDF1.1/1.3*), BS287 (*StCDF1.1/1.4*), Lishu6 (*StCDF1.1/1.1/1.4/1.5*), and Eshu5 (*StCDF1.1/1.1/1.4/1.6*), respectively. The endogenous promoter, full gene sequence, and terminator of *StCDF1.2*, *StCDF1.3*, *StCDF1.5*, and *StCDF1.6* were amplified using the primer StCDF1-TG-1. Because of SNPs in the *StCDF1.4* primer-binding region, its sequence was amplified using a different primer, StCDF1-TG-2. The purified amplification products were cloned into the PAMBIA2300 vector through homologous recombination. Transformation was performed using three-week-old plantlets. Agrobacterium-mediated transformation (Ye et al., 2018) was employed to introduce these constructs into Qingshu9 (*StCDF1.1/1.1/1.1/1.1*) background. Primer sequences are provided in Supplementary Table S3.

**Selection of *StCDF1* genotypes containing a single-copy mutant allele.**

Crosses were performed using Zhongshu3 (*StCDF1.1/1.1/1.3/1.4*), Jizhangshu12 (*StCDF1.1/1.1/1.2/1.4*), Lishu6 (*StCDF1.1/1.1/1.4/1.5*), and Eshu5 (*StCDF1.1/1.1/1.4/1.6*) as maternal parents, with C88 (*StCDF1.1/1.1/1.1/1.1*) as the paternal parent. For the F_1_ progeny of Jizhangshu12, the molecular markers StCDF1-shortinsert and StCDF1.4-p were used to select the genotypes *StCDF1.1/1.1/1.1/1.2* (containing *StCDF1.2* but lacking *StCDF1.4*). Additionally, StCDF1-shortinsert was used to screen for genotypes containing only *StCDF1.1/1.1/1.1/1.1*. For the F_1_ progeny of Zhongshu3, the markers StCDF1-shortinsert and StCDF1.3-insert were employed to identify the following genotypes: *StCDF1.1/1.1/1.1/1.1* (lacking *StCDF1.3* and *StCDF1.4*), *StCDF1.1/1.1/1.1/1.3* (containing *StCDF1.3* but lacking *StCDF1.4*), and *StCDF1.1/1.1/1.1/1.4* (containing *StCDF1.4* but lacking *StCDF1.3*). For the F_1_ progeny of Lishu6, StCDF1-shortinsert and StCDF1.4-p were used to identify *StCDF1.1/1.1/1.1/1.1* (lacking *StCDF1.4* and *StCDF1.5*) and *StCDF1.1/1.1/1.1/1.5* (containing *StCDF1.5* but not *StCDF1.4*). For the progeny of Eshu5, StCDF1-shortinsert and StCDF1.6-CAPS were used to identify the following genotypes: *StCDF1.1/1.1/1.1/1.1* (lacking *StCDF1.4* and *StCDF1.6*), *StCDF1.1/1.1/1.1/1.4* (containing *StCDF1.4* but lacking *StCDF1.6*), and *StCDF1.1/1.1/1.1/1.6* (containing *StCDF1.6* but lacking *StCDF1.4*). To identify *StCDF1.6* using the StCDF1.6-CAPS, PCR amplification was performed, followed by digestion of the amplification product with the BclI restriction enzyme (NEB) according to the manufacturer’s protocol, and analysis by polyacrylamide gel electrophoresis. The presence of 84 bp and 42 bp bands confirmed the presence of *StCDF1.6*. Primes sequences are listed in Supplementary Table S3.

**RNA extraction and quantitative real-time PCR.**

Transgenic lines harboring *StCDF1.2–1.6* and the wild type plants were cultivated under long-day conditions (16 h light/8 h dark) for three weeks. Samples were collected from the third fully expanded leaf from the apex of each plant at four-hour intervals, with three biological replicates taken at each time point. The collected leaf samples were immediately frozen in liquid nitrogen and stored at -80°C. Total RNA was extracted using the RNAprep Pure Plant Kit (TIANGEN BIOTECH, BEIJING) following the manufacturer’s protocol. Subsequently, 1 μg of total RNA was reverse transcribed into cDNA using the PrimeScript™ RT reagent Kit (TaKaRa) according to the manufacturer’s instructions. Quantitative real-time PCR (qRT-PCR) was performed using the Applied Biosystems StepOnePlus™ system. Primer sequences used for qRT-PCR are provided in Supplementary Table S3. Expression levels were normalized to the internal reference gene *StElF3e*. Each PCR reaction was conducted in triplicate. Relative gene expression levels were determined using the 2^-ΔΔCt^ method.

**Haploid induction technology for creating long-day-adapted diploid germplasm.**

The haploid inducer PL4 was crossed with Lishu6 (*StCDF1.1/1.1/1.4/1.5*) and Eshu5 (StCDF*1.1/1.1/1.4/1.6*) to obtain F_1_ progeny. F_1_ seeds without embryo spots were selected. The selected F_1_ seeds were sown in soil, and leaf DNA was extracted approximately one month later. Molecular markers StCDF1-shortinsert and StCDF1.4-p were employed to screen F_1_ individuals lacking *StCDF1.4* but containing *StCDF1.5*. Similarly, molecular markers StCDF1-shortinsert and StCDF1.6-CAPS were employed to screen F_1_ individuals lacking *StCDF1.6* but containing *StCDF1.4* and lacking *StCDF1.4* but containing *StCDF1.6*. The sequences of these molecular markers are listed in Supplementary Table S3. Flow cytometry was used to determine the ploidy level of the F_1_ plants as described previously (Zhang et al., 2022), and diploid germplasms with the genotypes *StCDF1.1/1.4*, *StCDF1.1/1.5* and *StCDF1.1/1.6* were selected.

**References**

Li, H., & Durbin, R. (2009) Fast and accurate short read alignment with Burrows-Wheeler transform. Bioinformatics, **25**, 1754–1760.

Li, H., Handsaker, B., Wysoker, A., Fennell, T., Ruan, J., Homer, N., Marth, G., Abecasis, G., Durbin, R., & 1000 Genome Project Data Processing Subgroup (2009) The Sequence Alignment/Map format and SAMtools. Bioinformatics, **25**, 2078–2079.

McKenna, A., Hanna, M., Banks, E., Sivachenko, A., Cibulskis, K., Kernytsky, A., Garimella, K., Altshuler, D., Gabriel, S., Daly, M., & DePristo, M. A. (2010) The Genome Analysis Toolkit: a MapReduce framework for analyzing next-generation DNA sequencing data. Genome research, **20**, 1297–1303.

Pham, G. M., Hamilton, J. P., Wood, J. C., Burke, J. T., Zhao, H., Vaillancourt, B., Ou, S., Jiang, J., & Buell, C. R. (2020) Construction of a chromosome-scale long-read reference genome assembly for potato. GigaScience, **9**, giaa100.

Ye, M., Peng, Z., Tang, D., Yang, Z., Li, D., Xu, Y., Zhang, C., & Huang, S. (2018) Generation of self-compatible diploid potato by knockout of S-RNase. Nature plants, **4**, 651–654.


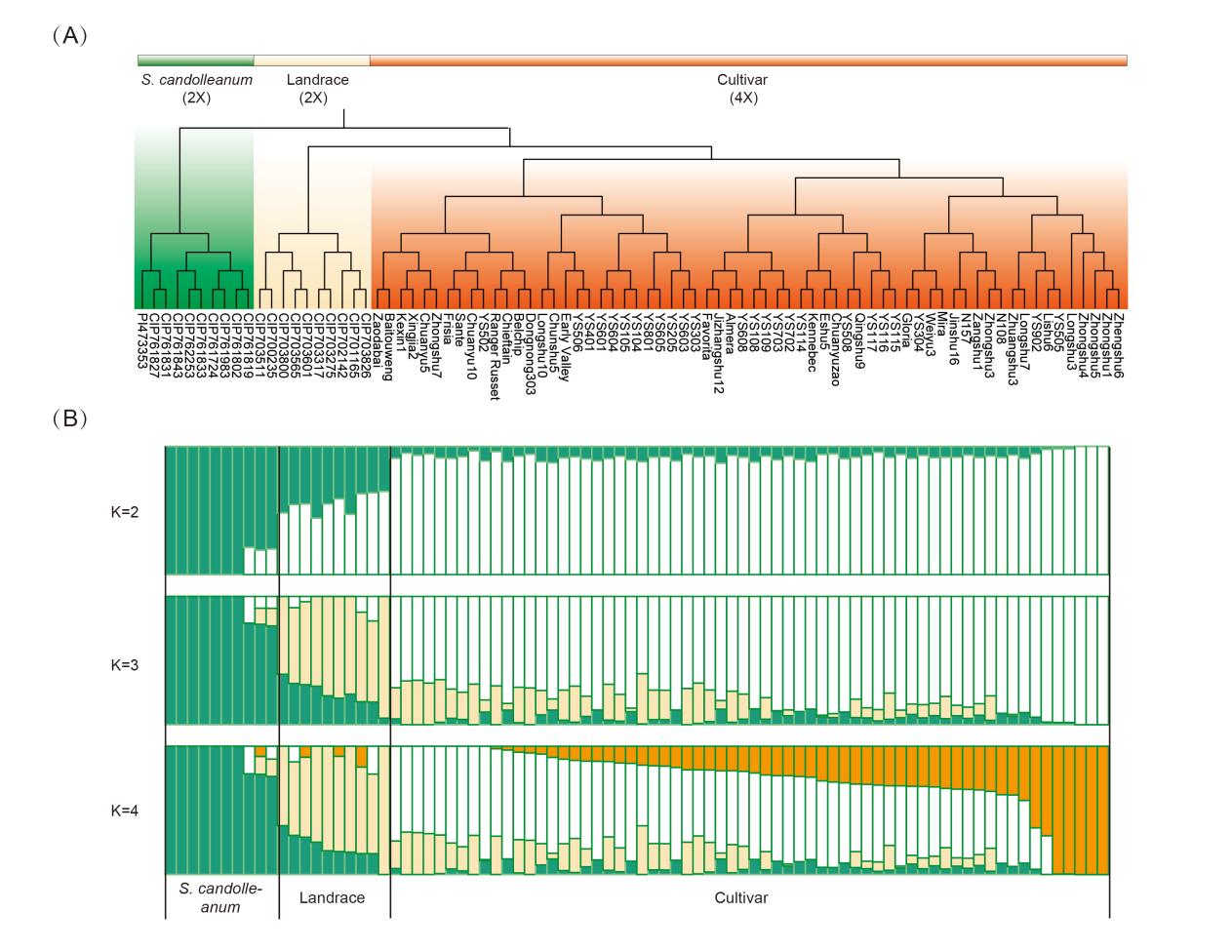


**Supplementary Figure S1.** Genomic diversity and population structure of the 84 potato accessions used in this study. (A) Phylogenetic tree 84 potato accessions, including 10 wild *Solanum candolleanum* accessions, 10 landraces, and 64 tetraploid cultivars. The maximum likelihood tree was constructed using 79,213 fourfold degenerate synonymous sites (4DTv). (B) Population structure analysis of the 84 potato accessions, with *K*-values of 2-4.


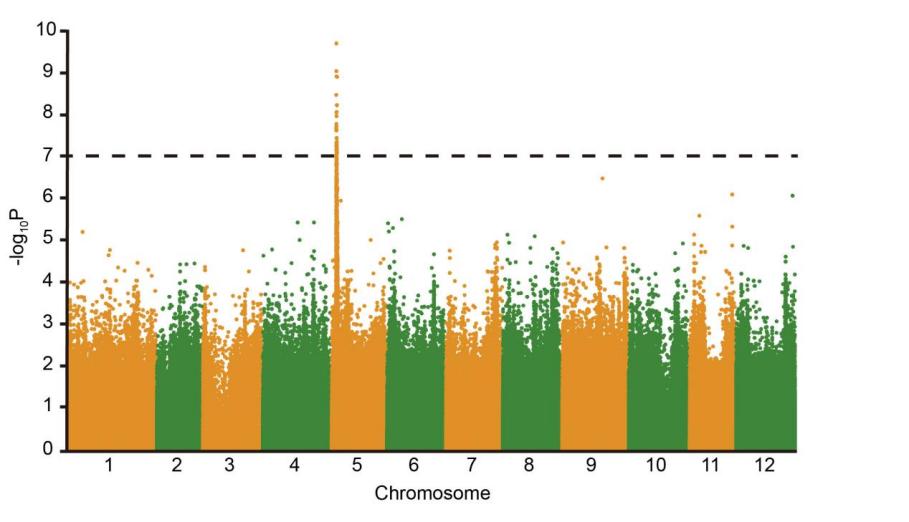


**Supplementary Figure S2.** Genome-wide association study of the day-to-maturity using 64 potato cultivars. The red arrow indicates the *StCDF1* locus, identified on chromosome 5.


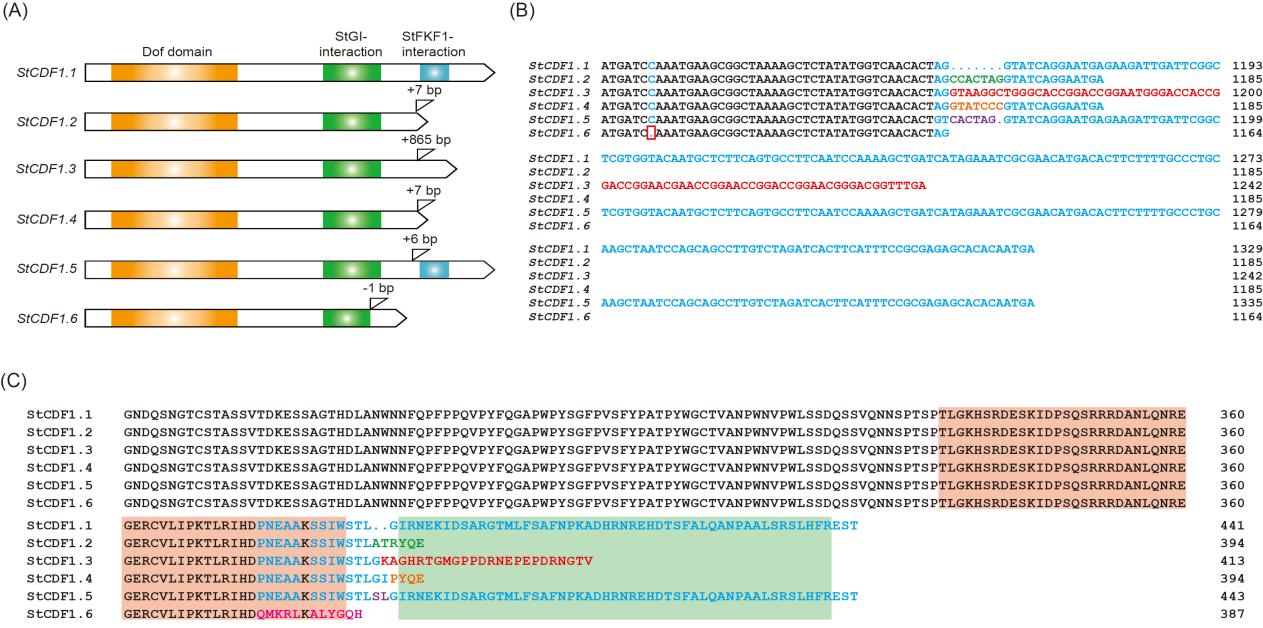


**Supplementary Figure S3.** Gene structures and sequences of *StCDF1.1*–*1.6*. (A) Gene structure of six *StCDF1* alleles. Orange shadow: structural domain in which StCDF1 interacts with StGI; Light green shadow: structural domain in which StCDF1 interacts with StFKF1. (B) The nucleotide sequence alignment of six *StCDF1* alleles at the variant points. (C) Amino acid sequence alignment of six *StCDF1* alleles.


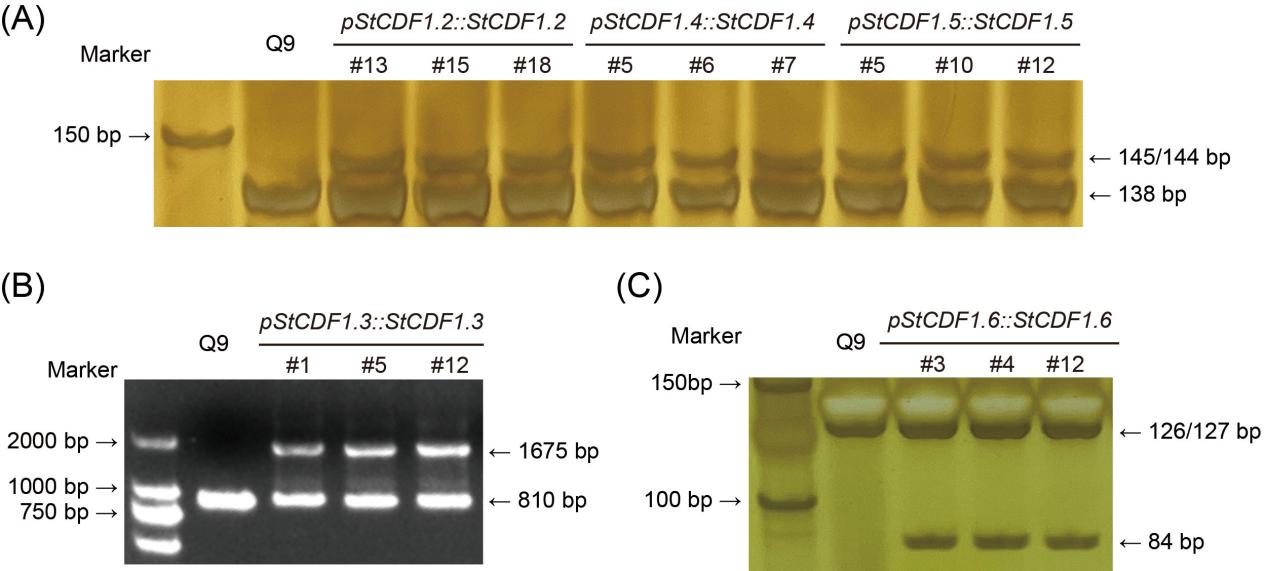


**Supplementary Figure S4.** Identification of *StCDF1* transgenic lines. (A) Identification of *StCDF1.2*, *StCDF1.4*, and *StCDF1.5* transgenic lines using primer StCDF1-shortinsert. The wild type (wt) showed a 138 bp band. Transgenic lines with *StCDF1.2* and *StCDF1.4* showed an additional 145 bp band due to a 7 bp insertion, while the *StCDF1.5* transgenic line showed an extra 144 bp band due to a 6 bp insertion. (B) Identification of *StCDF1.3* transgenic lines using primer StCDF1.3-insert. The wt shows an 810 bp band, while the *StCDF1.3* transgenic line displays an additional 1675 bp band due to an 865 bp insertion. (C) Identification of *StCDF1.6* transgenic lines using primer StCDF1.6-CAPS. The *StCDF1.6* transgenic line shows an 84 bp band after enzyme digestion, which is absent in wildtype (Q9). Primes sequences are listed in Supplementary Table S3.


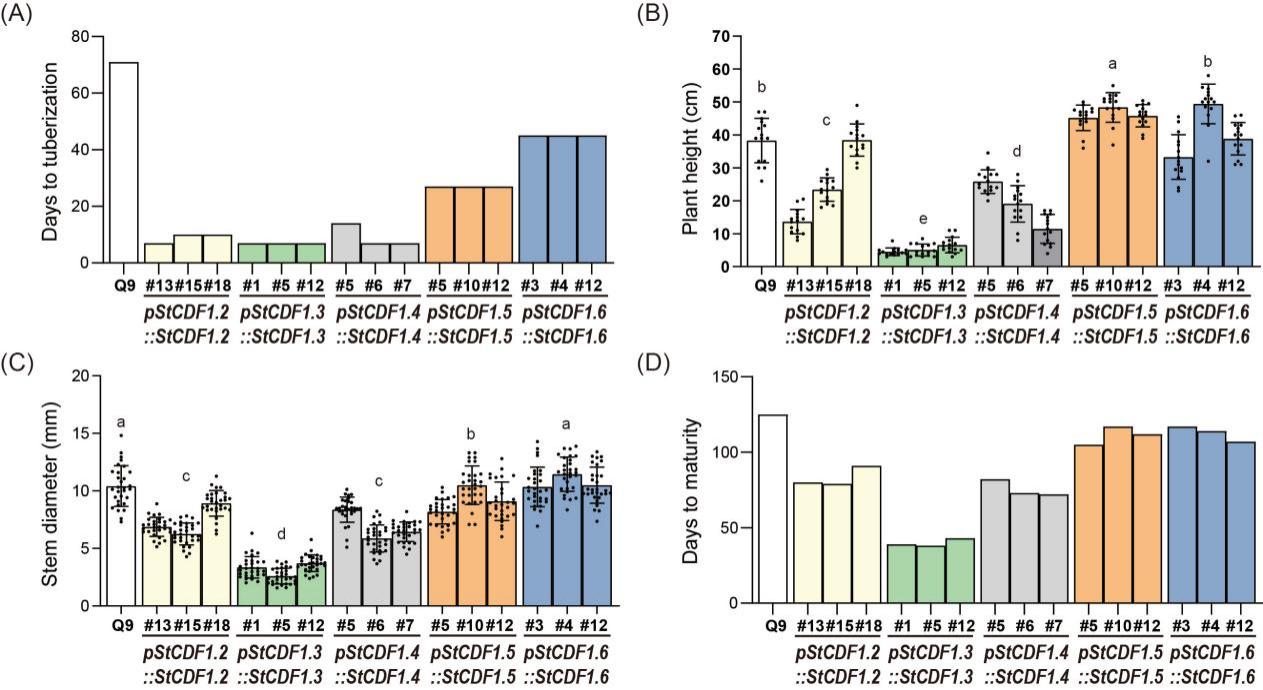


**Supplementary Figure S5.** Phenotypic analysis of the *StCDF1* transgenic lines in artificial long day condition. (A) Tuberization initiation time for the *StCDF1* transgenic lines under artificial long-day conditions (16 h light/8 h dark). (B) Plant height of the *StCDF1* transgenic lines after 45 d of cultivation under artificial long-day conditions (16 h light/8 h dark). The results are presented as means ± SD (*n* = 15 plants).

**Supplementary Figure S6.** Tuberization initiation time associated with each *StCDF1* allele in a tetraploid heterozygous background. Crosses were made using Zhongshu3 (*StCDF1.1/1.1/1.3/1.4*), Jizhangshu12 (*StCDF1.1/1.1/1.2/1.4*), Lishu6 (*StCDF1.1/1.1/1.4/1.5*), and Eshu5 (*StCDF1.1/1.1/1.4/1.6*) as maternal parents and C88 (*StCDF1.1/1.1/1.1/1.1*) as the paternal parent. Genotypes with single-copy mutant alleles were selected and cultivated under long-day conditions (16 h light/8 h dark). Initiation of tuberization was evaluated weekly, starting three weeks post-emergence, by recording tuberization rates for 10 plants per genotype.


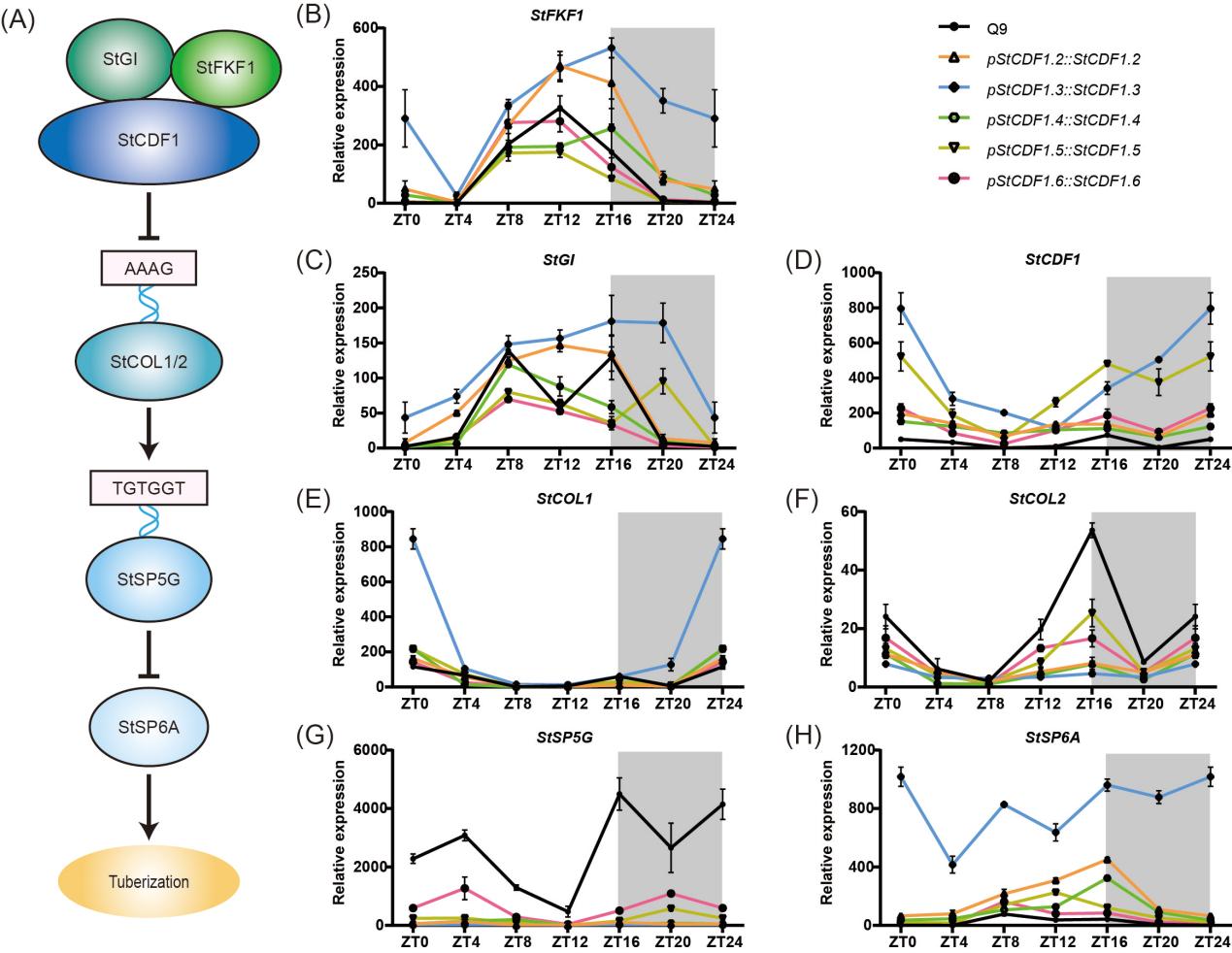


**Supplementary Figure S7.** Expression of genes related to the *StCDF1*-mediated photoperiod-regulated tuberization pathway. (A) *StCDF1*-mediated regulation of long-day tuberization. (B–H) Expression of *StFKF1*, *StGI*, *StCDF1*, *StCOL1*, *StCOL2*, *StSP5G*, and *StSP6A* in *StCDF1* transgenic lines under long-day conditions (16 h light/8 h dark).


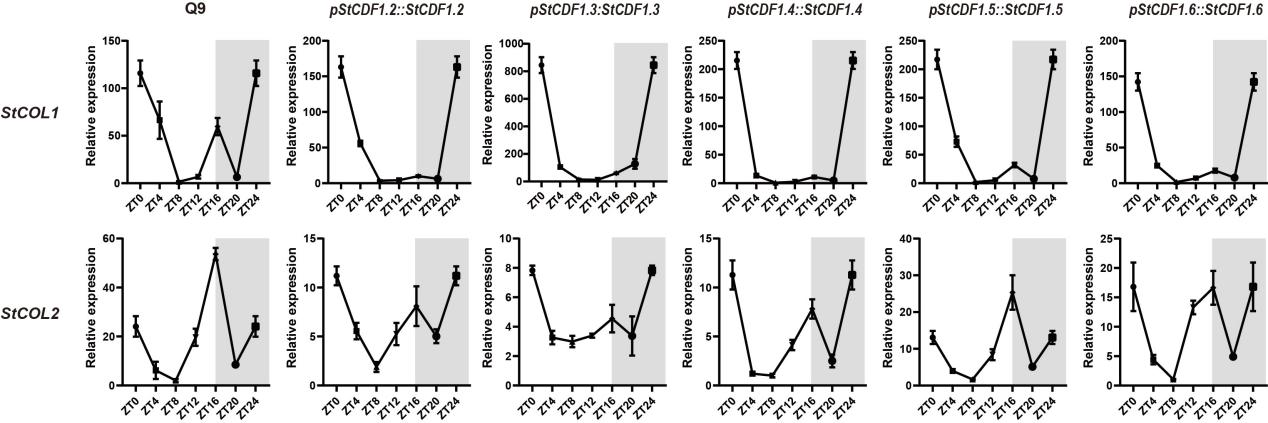


**Supplementary Figure S8.** Diurnal expression of *StCOL1* and *StCOL2* in the *StCDF1* transgenic lines under long-day conditions (16 h light/8 h dark).


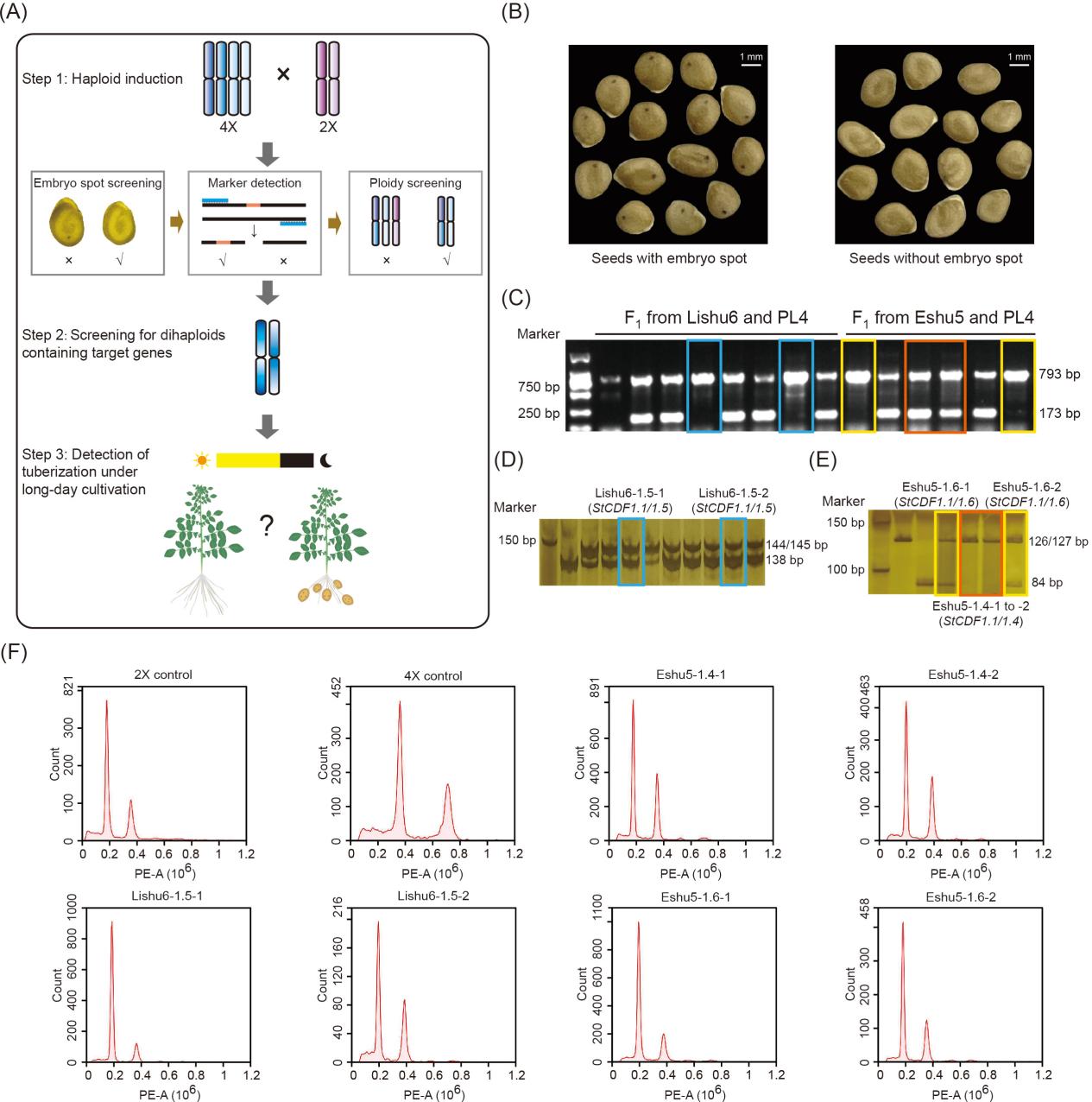


**Supplementary Figure S9.** Generation, via haploid-induction technology, of long-day adapted dihaploid lines containing *StCDF1.4*–*1.6*. (A) Induction of dihaploid lines from tetraploid potatoes. (B) Screening of seeds for embryos after haploid induction. (C–E) represent the genotypes screened for *StCDF1.4*, *StCDF1.5*, and *StCDF1.6*, respectively. Blue rectangles: *StCDF1.1/1.5* genotypes screened from the offspring of Lishu6 and PL4; yellow rectangles: *StCDF1.1/1.6* genotypes screened from the offspring of Eshu5 and PL4; orange rectangle: *StCDF1.1/1.4* genotypes screened from the offspring of Eshu5 and PL4. Primer sequences are listed in Supplementary Table S3. (F) Chromosome ploidy detection of the F_1_ individuals selected using molecular markers.
